# Nursing students’ knowledge and attitudes toward older adults

**DOI:** 10.3389/fpubh.2023.1150261

**Published:** 2023-10-09

**Authors:** Cidália Castro, Ricardo Antunes, Aida Simoes, Catarina Bernardes, Júlio Belo Fernandes

**Affiliations:** ^1^Egas Moniz Center for Interdisciplinary Research (CiiEM)Egas Moniz School of Health & Science, Almada, Portugal; ^2^Nurs* Lab, Almada, Portugal

**Keywords:** nursing student, older adult, aged, knowledge, attitude of health personnel, geriatric nursing, aging, public health

## Abstract

Nursing students, as the future healthcare workforce, hold immense potential in providing quality care to older adults and becoming advocates for promoting aging and public health, thus contributing significantly to addressing the multifaceted challenges of our aging society. Nurses’ knowledge and attitudes about aging affect health care quality. Negative and unattractive representations of the social problems associated with aging contaminate nursing students’ attitudes. Nursing schools are challenged to develop new curricula to prepare future nurses for the inherent complexity of an aging society. This study aims to assess the knowledge and attitudes of nursing students toward older adults and identify the variables that can influence these attitudes. Quantitative research was carried out through the application of an online survey using a cross-sectional descriptive research design. A total of 182 nursing students completed the online survey. Progression in the nursing course was statistically significant; the more students advanced, the more positive attitudes and knowledge they revealed about aging; 39% of students have daily contact with their grandparents; however, only 14.8% would like to work with older adults. Multiple linear regression revealed that the most important factor for positive attitudes and knowledge about aging was regular contact with grandparents, followed by progression in the nursing course. The students’ age was not a significant factor in improving attitudes or expanding knowledge regarding older adults. In a multidimensional logic, the deepening of knowledge about aging and the socialization of students with older adults are central factors that should reinforce curricula in nursing education.

## 1. Introduction

The demographic aging of the population is one of humanity’s most significant achievements, but it also constitutes a major challenge (1). It is estimated that by 2050 the population aged 60 or older is expected to increase from 962 million in 2017 to 2.1 billion in 2050 and 3.1 billion in 2100 (1). In the European Union, Portugal has the fourth-highest percentage age of older people (2), representing 23.4% of the total population (3). Aging can be a major problem if society is not prepared to deal with its own aging, presenting pejorative attitudes toward this phase of life (4). It is common to identify negative attitudes toward older adults in various sectors of society (4–6). These attitudes stem from ageism, which manifests in various forms, including stereotyping, prejudice, discrimination, marginalization, and disregard for older adults’ unique needs and contributions (7). The World Health Organization analysis of the World Values Survey has revealed the widespread prevalence of negative or ageist attitudes toward older adults (8). This comprehensive survey involved over 85,000 participants from 60 countries, uncovering significant variations in the roles assigned to older adults across different nations and cultures. These variations suggest that factors such as socioeconomic development, income inequality, the prominence of youth-oriented culture, and the breakdown of family support structures contribute to the levels of ageism observed in societies. Remarkably, the lowest levels of respect were reported in high-income countries.

The results of this analysis firmly establish the distressing reality of the commonality of ageism. Even more alarming is that most people are unaware of their subconscious stereotypes regarding older adults. These unconscious biases perpetuate ageism and impede efforts to foster inclusive and age-friendly societies (7). Furthermore, these negative attitudes are also prevalent within the healthcare sector itself (4, 6).

Older adults represent the leading group of users seeking healthcare and tend to have longer stays in hospitals, significantly impacting the overall financial costs of healthcare systems (9–11). There has been a growing commitment to developing healthcare models and strategies to implement care for older adults (12); however, healthcare systems continue to prioritize fulfilling the care needs of younger people while failing to address the complexity of older adults’ health concerns (13, 14).

Studies have shown that healthcare professionals’ negative attitudes toward aging lead to worse received care and poorer health outcomes for older adults (15). Among the professions, nurses in the healthcare field are the best positioned to meet the growing demands imposed on the healthcare system by an aging society (16).

Nursing schools are constantly challenged to develop curricula to prepare future nurses for managing, coordinating, and providing health care for older adults. Qualified nurses are needed to care for this complex population (17). Nurses’ knowledge about aging, as well as nurses’ representations and attitudes toward older adults, are considered a set of factors that affect the quality of health care (11).

Increasing knowledge about aging improves positive representations and attitudes toward older adults. Studies on nursing students’ attitudes toward older adults have shown an association between negative attitudes and students’ lack of interest in pursuing nursing careers in gerontology (17, 18). In addition, there is evidence that interactions with older adults can effectively decrease ageism. Thus, it is essential to promote intergenerational contact (19–21). There is an apparent necessity to enhance knowledge about aging and foster open, natural, and mutual relationships, which will ultimately reduce negative attitudes toward aging. The promotion of positive attitudes and the increase in nursing students’ knowledge about aging are intrinsically linked to the field of public health in the sense that they stimulate how these future health professionals contribute to health promotion, prevention, personalized health care, and encouraging the development of community health programs, outreach initiatives, and health education efforts designed to improve the overall health and well-being of older adults.

The different syllabi of nursing training affect students’ attitudes toward older adults (17, 22). Nursing students at more advanced levels of academic training have shown improved attitudes toward this population (18, 22, 23). However, some studies point to inconsistent results on how academic progression influences student attitudes. For example, Gould et al. (24) have shown that nursing students tended to have more negative attitudes toward older adults after the first clinical placement.

The present study aims to assess the knowledge and attitudes of nursing students toward older adults and identify the variables that can influence these attitudes.

## 2. Methods

### 2.1. Study design

This quantitative study was conducted using a cross-sectional descriptive web-based survey design.

### 2.2. Sampling and recruitment

Nursing students were recruited from a Portuguese private Higher School of Health in the region of Lisbon and Tagus Valley. We used a non-probabilistic convenience sample of 182 students. All undergraduate students attending the nursing degree course were invited to participate in the study through their school e-mail addresses.

The inclusion criteria encompassed all students enrolled in any of the 4 years of the nursing degree who accepted to participate in the study.

### 2.3. Data collection

Data collection was carried out by applying an online survey between February and May of 2022. The data collection instrument was written in Portuguese and included the study information page, psycho-socio-demographic questionnaire, Palmore facts and aging quiz (25, 26), and Kogan’s scale (27, 28). All questions were transcribed into Google Forms^™^. A pre-test was applied to 22 students, and a final online survey was conducted. The database was structured in four dimensions:

1. Sociodemographic and academic attributes: sex, age, academic levels.
2. Interaction and social relationships with older adults. This dimension was based on the regularity of contacts between students and their grandparents.
3. Knowledge concerning aging. Dimension assessed using the Palmore Facts and Aging Quiz, which consists of 25 questions with four answer choices where only one is correct.
4. Attitudes toward older adults. Dimension was measured using Kogan’s scale, which consists of 34 statements, where 17 measure negative attitudes and the other 17 measure positive attitudes. This scale ranges from “1” to the lowest agreement and “6” to the highest agreement. The negative items were inverted, so the final score indicated that the higher the value, the more positive the attitudes. Cronbach’s alpha of the scale was 0.764.

### 2.4. Data analysis

Statistical analysis was carried out using SPSS Version 26.0. First, descriptive statistics were calculated to characterize the sample and specify student attributes, and then an inferential statistics analysis was carried out. The evaluation of normality and homogeneity of variances were performed using the Kolmogorov–Smirnov test and the Levene test, respectively; the value of *p* < 0.05 was considered statistically significant. ANOVA and *post-hoc* analyses, using the Bonferroni test, were used to analyze the relationship between differences in attitudes and degree of knowledge about aging and the characteristics of students. Finally, a linear regression model was developed to assess the importance of different factors related to the Kogan scale scores.

### 2.5. Ethics and procedures

This study follows the principles of the Declaration of Helsinki. The study protocol was analyzed and approved by the Egas Moniz Higher School of Health Board of Directors and the Institutional Ethics Committee (Date: 30 April 2020; ID 882). The survey’s first page detailed the objectives and procedures of the study and stated that participation was entirely voluntary, and participants could decline to answer any questions. Participants had to accept and agree to the online informed consent to complete the survey. The study was conducted to guarantee the confidentiality and anonymity of data.

## 3. Results

A total of 182 nursing students completed the online survey (Table 1); most respondents were female (91.2%), with a mean age of 22.2 years (SD 4.99). The distribution of students by the 4 years of the nursing degree reveals a more equitable distribution, with a lower percentage of students in the 4th year (17.6%) and more expressive in the 3rd year (31.3%).

**Table 1:** Participants’ characteristics.

Variables ^1^	Total students *n* = 182	
	*n*	%
Sex		
Men	16	8.8
Women	166	91.2
Age		
18–20	71	39.0
21–23	74	40.7
> = 24	37	20.3
Year of graduation		
1°	48	26.4
2°	45	24.7
3°	57	31.3
4°	32	17.6
Regularity of contacts with grandparents		
Daily	71	39.0
Weekly	53	29.1
Monthly	15	8.2
Annually	15	8.2
Never	28	15.4
At what age group would you like to work in nursing?		
Older adults	27	14.8
Not with older adults	90	49.5
Do not know / Indifferent	65	35.7

^1^Presence of missing data.

In the dimension of sociability with older adults, 39% of the students reported having daily contact with their grandparents, 29.1% weekly contact, and 15.4% had no contact with grandparents. Finally, only 14.8% of students indicated that they would like to work with older adults.

Subsequently, an inferential analysis was performed (Table 2) in which the dependent variables were attitudes toward older adults, measured by the Kogan’s scale, which ranges from 1 to 6, and the degree of knowledge about aging, measured by the Palmore Facts and Aging Quiz’s scale, which ranges from 0 to 25.

**Table 2:** Kogan’s scale and aging knowledge index in articulation with students’ characteristics.

	Kogan’s scale (attitudes) (range 1–6)		Palmore facts and aging quiz’s scale (range 0–25)	
	Mean (SD)	*p-value*	Mean (SD)	*P-value*
Sex				
Men	4.07 (0.43)	0.487^a^	11.81 (1.76)	0.672^a^
Women	4.17 (0.45)		11.46 (2.42)	
Age				
18 to 20	4.14 (0.44)	0.367^b^	11.18 (2.45)	0.109^b^
21–23	4.20 (0.45)		11.89 (2.37)	
> = 24	4.11 (0.45)		11.27 (2.14)	
Year of graduation				
1°	4.11 (0.46)	0.004	10.71 (2.65)	0.003
2°	4.01 (0.47)		10.96 (1.89)	
3°	4.26 (0.41)		11.75 (2.14)	
4°	4.33 (0.29)		12.94 (2,24)	
Regularity of contacts with grandparents				
Daily	4.26 (0.47)	0.002	12.08 (2.07)	0.035
Weekly	4.19 (0.44)		11.61 (2.66)	
Monthly	4.15 (0.42)		11.33 (2.46)	
Annually	4.11 (0.39)		10.80 (3.04)	
Never	3.88 (0.36)		10.23 (1.88)	
At what age group would you like to work in nursing?				
Older adults	4.24 (0.43)	0.571	12.26 (2.26)	0.125
Not with older adults	4.14 (0.45)		11.50 (2.16)	
Do not know / Indifferent	4.17 (0.44)		11.15 (2.63)	

^a^Mann–Whitney U’s test;^b^Kruskal-Wallis’s test.

The variables sex and age group did not present a normal distribution; therefore, non-parametric tests were used: the Mann–Whitney U test and the Kruskal-Wallis test, respectively. Both variables, sex and age groups, were not statistically significant in relation to differences in attitudes toward older adults or the degree of knowledge about aging. The remaining variables exhibited a normal distribution and homogeneity, and ANOVA tests were conducted. The statistical analysis indicated a significant relationship between Kogan scale values and progression in the nursing course (*p* = 0.004), indicating that as students’ progress in the course, there is an improvement in their attitudes. However, the attitudes of 2nd-year nursing students (M = 4.01, SD = 0.47) were less positive than those of 1st-year students. *Post-hoc* analyses using the Bonferroni test showed no significant mean differences between 1st and 2nd-year students (*p* = 0.613). Still, significant differences were observed between 2nd and 3rd-year students (*p* = 0.016) and between 2nd and 4th-year students (*p* = 0.003).

Furthermore, the progression in the nursing course was associated with a clear increase in knowledge about older adults (*p* = 0.003). The *post-hoc* analysis, using the Bonferroni test, revealed statistically significant differences between 1st and 4th-year students (*p* = 0.001) and between 2nd and 4th-year students (*p* = 0.002).

Additionally, the regularity of contact with grandparents was also found to be statistically significant. The study showed that the more frequent the contact between students and their grandparents, the more positive their attitudes (*p* = 0.002) and the greater their knowledge about aging (*p* = 0.035).

Finally, in relation to the work contexts desired by the students, there was no statistically significant between the hypothetical dominant age group in the future workplaces and the differences in their attitudes (*p* = 0.571) or in the degree of knowledge (*p* = 0.125).

In Table 3, we aimed to examine the factors influencing the variation in students’ attitudes and knowledge about older adults. A multivariate analysis was conducted using a linear regression model to achieve this. Before the analysis, all assumptions required for linear regression were carefully checked. Two dependent variables were considered: the Kogan scale and the Palmore facts and aging quiz scale.

**Table 3:** Multivariate regression analysis considering Kogan’s scale and Palmore facts and aging quiz’s scale as dependent variable.

	*B*	β	*t*		*p* value	95% CI
Panel 1 Kogan’s scale as dependent variable						
Age	0.002	0.020	0.255		0.799	−0.01; 0.02
Year of graduation	0.077	0.179*	2.443		0.016	0.02; 0.14
Contact with grandparents	0.066	0.208*	2.610		0.010	0.02; 0.12
Palmore Facts and Aging Quiz’s scale.	0.033	0.173*	2.291		0.023	0.01; 0.06
Adjusted *R*^2^				0.123**		
*F* (4.177)				7.345**		
Panel 2 Palmore facts and aging quiz’s scale as dependent variable						
Age	0.028	0.058	0.760		0.448	−0.04; 0.10
Year of graduation	0.388	0.171*	2.377		0.019	0.07; 0.71
Contact with grandparents	0.468	0.282**	3.658		<0.001	0.22; 0.72
Kogan’s scale	0.877	0.167*	2.291		0.023	0.12; 1.63
Adjusted *R*^2^				0.153**		
F (4.177)				9.150**		

*p < 0.05; ** *p* < 0.001.

The analysis revealed that regular contact with grandparents was the most significant factor contributing to increased positive attitudes (*β* = 0.208; *p* = 0.010). The second most influential factor was the year of graduation, indicating that as students progressed in the nursing course, their positive attitudes toward older adults improved (*β* = 0.179; *p* = 0.016). The level of knowledge about aging represented the third explanatory factor; the better the knowledge about aging, the better the positive attitudes toward older adults (*β* = 0.173; *p* = 0.023). The age of nursing students did not significantly contribute to improving attitudes toward older adults (*β* = 0.020; *p* = 0.799).

Regarding the Palmore facts and aging quiz scale as the dependent variable, contact with grandparents emerged as the strongest predictor (*β* = 0.282; *p* < 0.001). Therefore, the more regular the contact with grandparents, the greater the knowledge about older adults. Additionally, the progression in the student’s year was associated with better knowledge (*β* = 0.171; *p* = 0.019), and positive attitudes toward older adults was also linked to better knowledge about aging (*β* = 0.167; *p* = 0.023). Age was not a significant factor in improving knowledge (*β* = 0.020; *p* = 0.799) in this model.

## 4. Discussion

The current study examined nursing students’ knowledge and attitudes toward older adults. A first general conclusion revealed that, in the comparison between these two dimensions of analysis, attitudes toward older adults were positively better placed than knowledge about aging.

The scale used in this study to assess knowledge about aging, the Palmore quiz, is based on a multidimensional logic. A more detailed analysis of the student’s responses on this scale made it possible to perceive that the need for learning about aging must go beyond the biological dimensions. The reinforcement of teaching should include learning in the psychological, social, and anthropological dimensions of aging. As previous studies also defend (17, 29), it is necessary to rethink the curricula and guide the syllabus to improve students’ knowledge and avoid prejudice, stereotyping, and discrimination. As was also shown, increasing knowledge leads to improved attitudes toward older adults.

A second general conclusion revealed the importance of sociability relationships with grandparents positively influencing attitudes and knowledge about aging. Studies on aging in Portugal point to this vital family dimension that provides solidarity networks between older adults and other family members (30). In this investigation, their solid social relationships with their grandparents stood out in the characterization of the students. 39% of the students reported having daily contact with their grandparents. These relationships involved regular interaction and periods of living with them. Most students (68.1%) had regular contact with one of their grandparents at least once a week. The findings indicated that this sociability was the main factor in improving attitudes and increasing knowledge about aging.

On the other hand, and in line with previous studies carried out in the field of nursing (31, 32), this study confirmed that health services whose population is mainly associated with older adults did not constitute an attractive work context for nursing students. Only 14.8% of the students in this study would like to work in health services with mostly older users. The media stereotype older adults in an unattractive and problematic representation, contaminating young people’s representations, including nursing students (17).

The findings of this study revealed that nursing students, at the beginning of the course, demonstrated less positive attitudes toward older adults. It is expected that nursing education will be able to change attitudes in a positive direction toward older adults. In this dimension, associated with nursing education, it was clear that the progression in the nursing course, in line with the results of Hernández et al. (22), improved the students’ attitudes. Despite these general results, this study also showed that the relationship between progression in the course and the improvement of attitudes was not linear. Previous studies have shown that working with older adults in contexts of greater institutionalization tends to have a negative impact on attitudes toward this population (22). Attitudes are associated with the type of experiences, positive or negative, that students have in contact with older adults; bad experiences reflect worse attitudes and representations (33). In the nursing course, the theoretical and the practical learning internship play a differentiated role that deserves a more detailed analysis.

This investigation was carried out in a university where the nursing course has a clinical placement in the 1st year. The findings pointed out that the 2nd year students showed a slight worsening in attitudes toward older adults after this first clinical placement, in line with the research of Gould et al. (24). An analysis of the school curriculum allowed us to verify that the 1st year students who answered this questionnaire did so before their first clinical placement. At the end of the 1st year, the first clinical placement was carried out in nursing homes. In addition, as it was the 1st year, students were privileged with learning dimensions centered on communication and providing hygiene and comfort care. On the other hand, the 2nd year students who responded to this survey mainly had the experience of clinical placements with institutionalized older adults in a context of less autonomous practice. The less positive results obtained with 2nd year nursing students might be attributed to these factors. Subsequently, as demonstrated by students’ progress and increased contact with older adults in less institutionalized contexts, whether in hospitals or community health, students, naturally associated with an increasing degree of technical and scientific autonomy, demonstrated better attitudes toward older adults. Progression in the following 3rd and 4th years showed that attitudes were continuously improving with progression in the course, which included longer clinical placements as one advanced in the degree. Another result that highlights the positive effect of academics has become evident in the relationship between age and progression in nursing courses. Although older students are in more advanced years of the nursing course, what was evidenced in this study was that age did not constitute an explanatory factor for the improvement of attitudes. Age was not significantly associated with either improved attitudes toward older adults or better knowledge of aging.

### 4.1. Strengths and limitations

This study allowed us to highlight the importance of an analysis carried out in different years of the nursing course, demonstrating how attitudes do not follow linearity. This fact encourages the realization of longitudinal studies where the theoretical and practical components typical of nursing education are articulated.

We consider that this study had some limitations that deserve to be mentioned: first, the sample’s limited size and composition, the disproportion that usually exists between male and female students in nursing require a larger sample; second, its limitation to only one nursing school, despite the diversity of the students’ origins, they all come from the Lisbon metropolitan area, in this sense, the results represent a population of students from urban areas, which limits possible generalizations to other contexts and settings; third, due to social desirability, the participants’ answers in the self-report survey may not represent their actual behaviors. However, we believe that the online nature of the survey and since no identifying data were collected, these limitations have been mitigated.

## 5. Conclusion

The students in this investigation had a past and present history of regular contact with older adults. This socialization was central in forming representations and positive attitudes toward older adults. In fact, despite the benefits provided by the solidarity and reciprocity developed between family members, it is essential to mention that such internal similarity makes it challenging to include members outside the family context in these networks and that it also goes hand in hand with a deficit of public support institutions. If this dimension linked to the quotidian interaction with older adults represented the positive pole in this investigation, on the negative side, we would highlight the insufficient knowledge and the little attractiveness that the theme and the contexts of working with an older population still constitute for nursing students. The field of care in gerontology could be more attractive to nursing students. Only 14.8% expressed the desire to work with older adults. However, the study showed that sociability with older adults and increased knowledge improve attitudes toward this population. The recommendations of this study go toward intensifying the inclusion of theoretical disciplines associated with the aging phenomenon; the diversification of clinical placements with older adults, including professional, recreational, cultural, or sporting associations geared toward older adults; the creation of volunteering programs in articulation with the faculty and associations in the community, in which the intention is to show the diversity of work that exists with older adults. Finally, to deepen knowledge at a more macro level, developing advanced levels of postgraduate studies and recognition by the Order of Nurses of the nursing specialty in Gerontology is recommended. Nursing students with positive attitudes toward aging and a solid knowledge base in this area are best positioned to contribute significantly to public health efforts. Their roles may encompass health promotion, personalized care, community programs, awareness initiatives, and health education, all with the goal of improving the health and well-being of seniors in their communities.

## Data availability statement

The data analyzed in this study is subject to the following licenses/restrictions: The data that support the findings of this study are available on request from the corresponding author. Requests to access these datasets should be directed to juliobelo01@gmail.com.

## Ethics statement

The studies involving humans were approved by Egas Moniz Higher School of Health and the Institutional Ethics Committee. The studies were conducted in accordance with the local legislation and institutional requirements. The participants provided their written informed consent to participate in this study.

## Author contributions

CC: conceptualization, data curation, formal analysis, investigation, methodology, writing, and reviewing. RA: formal analysis, investigation, methodology, writing, and reviewing. AS and CB: conceptualization, investigation, methodology, writing, and reviewing. JF: conceptualization, data curation, formal analysis, investigation, methodology, writing, reviewing and editing, and project administration. All authors contributed to the article and approved the submitted version.
